# The ameliorating effect of withaferin A on high-fat diet-induced non-alcoholic fatty liver disease by acting as an LXR/FXR dual receptor activator

**DOI:** 10.3389/fphar.2023.1135952

**Published:** 2023-02-23

**Authors:** Varsha D. Shiragannavar, Nirmala G. Sannappa Gowda, Lakshana D. Puttahanumantharayappa, Shreyas H. Karunakara, Smitha Bhat, Shashanka K. Prasad, Divya P. Kumar, Prasanna K. Santhekadur

**Affiliations:** ^1^ Department of Biochemistry, Center of Excellence in Molecular Biology and Regenerative Medicine, JSS Medical College, JSS Academy of Higher Education and Research, Mysore, India; ^2^ Department of Biotechnology and Bioinformatics, JSS Academy of Higher Education and Research, Mysore, Karnataka, India; ^3^ Bioactive Compound Laboratory, Faculty of Agriculture, Chiang Mai University, Chiang Mai, Thailand

**Keywords:** inflammation, fibrosis, withaferin A, fatty liver, liver X receptor-α, farnesoid X receptor

## Abstract

**Introduction:** Non-alcoholic fatty liver disease (NAFLD) incidence has been rapidly increasing, and it has emerged as one of the major diseases of the modern world. NAFLD constitutes a simple fatty liver to chronic non-alcoholic steatohepatitis (NASH), which often leads to liver fibrosis or cirrhosis, a serious health condition with limited treatment options. Many a time, NAFLD progresses to fatal hepatocellular carcinoma (HCC). Nuclear receptors (NRs), such as liver X receptor-α (LXR-α) and closely associated farnesoid X receptor (FXR), are ligand-inducible transcription factors that regulate various metabolism-associated gene expressions and repression and play a major role in controlling the pathophysiology of the human liver. Withaferin A is a multifaceted and potent natural dietary compound with huge beneficial properties and plays a vital role as an anti-inflammatory molecule.

**Methods:** *In vivo*: Swill albino mice were fed with western diet and sugar water (WDSW) for 12, 16, and 20 weeks with suitable controls. Post necropsy, liver enzymes (AST, ALT, and ALP) and lipid profile were measured by commercially available kits using a semi-auto analyzer in serum samples. Liver histology was assessed using H&E and MTS stains to check the inflammation and fibrosis, respectively, using paraffin-embedded sections and mRNA expressions of these markers were measured using qRT-PCR method. TGF-β1 levels in serum samples were quantified by ELISA. *In vitro*: Steatosis was induced in HepG2 and Huh7 cells using free fatty acids [Sodium Palmitate (SP) and Oleate (OA)]. After induction, the cells were treated with Withaferin A in dose-dependent manner (1, 2.5, and 5 μM, respectively). *In vitro* steatosis was confirmed by Oil-Red-O staining. Molecular Docking: Studies were conducted using Auto Dock Vina software to check the binding affinity of Withaferin-A to LXR-α and FXR.

**Results:** We explored the dual receptor-activating nature of Withaferin A using docking studies, which potently improves high-fat diet-induced NAFLD in mice and suppresses diet-induced hepatic inflammation and liver fibrosis *via* LXR/FXR. Our *in vitro* studies also indicated that Withaferin A inhibits lipid droplet accumulation in sodium palmitate and oleate-treated HepG2 and Huh7 cells, which may occur through LXR-α and FXR-mediated signaling pathways. Withaferin A is a known inhibitor of NF-κB-mediated inflammation. Intriguingly, both LXR-α and FXR activation inhibits inflammation and fibrosis by negatively regulating NF-κB. Additionally, Withaferin A treatment significantly inhibited TGF-β-induced gene expression, which contributes to reduced hepatic fibrosis.

**Discussion:** Thus, the LXR/ FXR dual receptor activator Withaferin A improves both NAFLD-associated liver inflammation and fibrosis in mouse models and under *in vitro* conditions, which makes Withaferin A a possibly potent pharmacological and therapeutic agent for the treatment of diet-induced NAFLD.

## Introduction

Non-alcoholic fatty liver disease (NAFLD) is an intricate malady that starts from steatosis (the accumulation of fats) and develops into non-alcoholic steatohepatitis (NASH) (Younossi et al., 2019). It constitutes early-stage inflammation and late-stage fibrosis, which leads to severe and irreversible terminal-stage hepatic complications like cirrhosis and malignancy of the liver, which is called hepatocellular carcinoma (HCC) (Allen et al., 2018). NAFLD is a significant risk factor for metabolic syndrome, which includes obesity and frequently associated co-maladies like type 2 diabetes mellitus (T2DM), cardiovascular disease (CVD), and other closely associated disorders (Byrne and Targher, 2015). NAFLD is remediable in the preliminary stages and can be addressed through lifestyle changes and medical help (Zeng et al., 2021). A lack of specific symptoms, knowledge, and awareness about the disease among patients makes the early-stage detection of NAFLD challenging (Cleveland et al., 2019; Busetto et al., 2021). In addition, there are few approved and available drugs for NAFLD on the market and they possess many side effects (Takahashi et al., 2015; Bhave and Ho, 2021). Therefore, looking for natural dietary compounds with minimal or no side effects for NAFLD treatment is greatly needed.

The pathogenesis of NAFLD is mainly linked to the excess and abnormal accumulation of free fatty acids (FFAs) in liver cells due to insulin resistance. It elevates the usage and production of FFAs in the liver and sensitizes hepatocytes to oxidative stress, mitochondrial dysfunction, and endoplasmic reticulum stress (ER stress), which further triggers various transcription factors and induces the secretion of TNF-α, TGF-β, MCP1, and the production of other closely associated inflammatory cytokines (Nascè et al., 2022; Akbari et al., 2021). This triggers macrophage recruitment and activates hepatic star-like cells or commonly known stellate cells (HSCs) and results in liver inflammation and fibrosis (Oates et al., 2019). Targeting the inflammation and fibrosis-associated transcription factors and their target genes and cytokines aids in NAFLD treatment.

Cellular nuclear receptors act as potential therapeutic targets for various clinical conditions like type 2 diabetes and NAFLD (Ballestri et al., 2016). Liver X receptor alpha (LXRα, NR1H3) plays a major role in regulating liver diseases (Tanaka et al., 2017). An associate of ligand-activated nuclear receptor-related transcription factors and the bile acid-binding nuclear receptor farnesoid X receptor (FXR) also has a potential function in human metabolism and an ameliorating effect against NAFLD (Ali et al., 2015). Obeticholic acid (OCA), which is a well-known bile acid and accepted drug for NASH, is known to significantly improve liver fibrosis *via* FXR (Younossi et al., 2022). In our recent study, we showed that withaferin A, which is a multifaceted drug from the ashwagandha plant, has an inhibitory effect on hepatocellular carcinoma *via* LXR-α activation and inhibits the NF-κB transcription factor (Santhekadur, 2017; Shiragannavar et al., 2021; Shiragannavar et al., 2022).

Our study indicates that withaferin A suppresses high-fat diet-induced metabolic features, NASH, and fibrosis acting as a ligand for both LXR-α and FXR. This study supports that the dual LXR/FXR activating nature of withaferin A could be used in treating human NASH.

## Materials and methods

### Materials

Withaferin A was purchased from Xenon Biosciences (India), and the chow diet was procured from Adita Biosys Pvt Ltd. The high-fat diet was procured from VRK Nutritional Solutions, India. Glucose and fructose were procured from Sisco Research Laboratories Pvt. Ltd. Commonly used liver function test enzymes, like aspartate transaminases or aspartate aminotransferase (AST), alanine transaminase or alanine aminotransferase (ALT), and alkaline phosphatase (ALP), and lipid molecules like total cholesterol, triglycerides (TG), and high-density lipoproteins (HDL) kits were purchased from Agape Diagnostics Ltd. TRIzol reagent, sodium palmitate, oleate and Oil Red O stain solution were purchased from Sigma Aldrich, St. Louis, Missouri, United States. cDNA synthesis and SYBR green kits were purchased from Thermo Fisher Scientific. A TGF-β1 ELISA kit was purchased from Krishgen Biosystems. HepG2 and Huh7 cells were purchased from NCCS Pune, India. The cell culture media Minimum Essential Medium Eagle (MEM), Ham DMEM/F-12, 1:1 mixture, bovine serum albumin, and hematoxylin were purchased from HiMedia, India. Fetal bovine serum (FBS) and antibiotics like penicillin/streptomycin were purchased from Gibco. 25-Hydroxycholesterol was a gift provided by Dr. Perumal Madan Kumar, CSIR-CFTRI, Mysuru. Taurochenodeoxycholic acid and deoxycholic acid were a gift from Dr. Ramprasad Talahalli, CSIR-CFTRI, Mysuru.

### Diet composition

Male Swiss albino mice (4–6 weeks old) were fed *ad libitum* sugar water (SW) containing glucose (18.9 g/L) and fructose (23.1 g/L) and a high-fat diet (Western Diet, WD) containing 42% kcal from fat and 0.1% cholesterol. A standard chow diet and normal water were given to the control mice.

### Experimental animals and study design

All animal experiments were conducted following the ethical clearance and approval from the Jagadguru Sri Shivarathreeshwara Academy of Higher Education and the Research Institutional animal ethics committee (JSSAHER/CPT/IAEC/019/2020), JSS AHER, Mysore, Karnataka, India. In this study, male Swiss albino mice (weighting 15–20 g) were selected and separated equally into five groups of six animals as follows: Group 1: control, standard chow diet, and normal water (CDNW); Group 2: western diet (high-fat diet) and sugar water (WDSW); Group 3: WDSW with withaferin A up to 12 weeks (treatment of withaferin A from 8 to 12 weeks); Group 4: WDSW with withaferin A up to 16 weeks (treatment of withaferin A from 8 to 16 weeks); and Group 5: WDSW with withaferin A up to 20 weeks (treatment of withaferin A from 8 to 20 weeks). Every 3 days, the treatment groups received withaferin A (1.25 mg/kg body weight, DMSO 0.1%), while the control group received DMSO (0.1%) intraperitoneally before the dark cycle of each day. To compare the treatment groups, Group 1—CDNW and Group 2—WDSW mice also received diet specifications, as previously mentioned for a duration of 12, 16, and 20 weeks. Briefly, Group 3 mice [mice that received WDSW (until 12 weeks) + withaferin A treatment for 4 weeks (from the 8^th^ week until the 12^th^ week)] were compared with the mice group that received CDNW and WDSW for 12 weeks. Group 4 mice [mice that received WDSW (16 weeks) + Withaferin A treatment for 8 weeks (from the 8th week until the 16th week)] were compared with the mice group that received CDNW and WDSW for 16 weeks. Group 5 mice [mice that received WDSW (20 weeks) + Withaferin A treatment for 12 weeks (from the 8th week until the 20^th^ week)] were compared with the mice group that received CDNW and WDSW for 20 weeks. Furthermore, images of individual groups of mice that received the WDSW + Withaferin A treatment for 12, 16, and 20 weeks along with their corresponding controls and WDSW groups are depicted in Supplementary Figures S6A–C.

### Serum biochemical measurements

Hepatic function enzymes, such as ALT, AST, and ALP, and the lipid profile comprising triglycerides, cholesterol, and HDL were determined using commercially available kits and is based on the associated manuals (Agappe Diagnostics Ltd.). The traditional GOD-POD method was used to measure the level of serum glucose. LDL was calculated using a simple mathematical formula as follows:

LDL = Total cholesterol–HDL–Triglycerides/5

### Histopathological estimation

Mice were sacrificed at the following time intervals (12, 16, and 20 weeks) to collect the liver tissue. The tissue samples were immediately formalin-fixed and stored at room temperature for subsequent processing and embedded by a standard technique in paraffin blocks for future usage. H&E and Trichome Masson’s (TMS) staining were performed to assess inflammation and visualize fibrosis, respectively.

### Quantitative polymerase chain reaction

The liver tissue was preserved at −80 °C. The TRIzol reagent (Thermo Fisher Scientific) was used to extract total RNA from frozen livers. The verso cDNA synthesis kit was used to create cDNA from 1 μg of total RNA. qPCR was performed on a Rotor-Gene Q (Qiagen) PCR system using the SYBR green kit. These qPCR results were expressed as a fold change relative to the control group, and values were normalized to β-actin mRNAs. The primer sequences used in our experiments were as follows:

TNF-α forward: 5′-ATG​GCC​TCC​CTC​TCA​TCA​GT-3′

TNF-α reverse: 5′-TTT​GCT​ACG​ACG​TGG​GCT​AC-3′

IL-6 forward: 5′-GTC​CTT​CCT​ACC​CCA​ATT​TCC​A-3′

IL-6 reverse: 5′-TAA​CGC​ACT​AGG​TTT​GCC​GA-3′

IL-1β forward: 5′-TGC​CAC​CTT​TTG​ACA​GTG​ATG-3′

IL-1β reverse: 5′-AAG​GTC​CAC​GGG​AAA​GAC​AC-3′

MCP1 forward: 5′-AGG​TGT​CCC​AAA​GAA​GCT​GT-3′

MCP1 reverse: 5′-AAG​ACC​TTA​GGG​CAG​ATG​CAG-3′

COL1A1 forward: 5′-CGA​TGG​ATT​CCC​GTT​CGA​GT-3′

COL1A1 reverse: 5′-GCT​GTA​GGT​GAA​GCG​ACT​GT-3′

COL3A1 forward: 5′-GAG​GAA​TGG​GTG​GCT​ATC​CG-3′

COL3A1 reverse: 5′-TTG​CGT​CCA​TCA​AAG​CCT​CT-3′

α-SMA forward: 5′-GCC​GAG​ATC​TCA​CCG​ACT​AC-3′

α-SMA reverse: 5′-ATA​GGT​GGT​TTC​GTG​GAT​GC-3′

β-actin forward: 5′-TGG​ATC​AGC​AAG​CAG​GAG​TAT​G-3′

β-actin reverse: 5′-GCA​TTT​GCG​GTG​GAC​GAT-3′

### ELISA for serum TGF-β1

An ELISA kit (Krishgen Biosystems) was used to quantify TGF-β1 in the plasma from the study animals following the manufacturer’s instructions. The results are presented as pg/mL after sample values were calculated using a standard curve (created by serially diluting known standards).

### Cell culture

Human hepatoma cells, namely, HepG2 and Huh7 cell lines, were cultured in MEM medium and Ham DMEM/F-12, 1:1 mixture, respectively, complemented with 10% penicillin (100 U/mL)/streptomycin (100 mg/mL) antibiotics in a humid incubator with 5% CO_2_ at 37 °C.

### *In vitro* steatosis induction

Stock solutions of sodium palmitate (SP) and oleate (OA) (Sigma-Aldrich, United States) were prepared, as previously described (Römer et al., 2021; Cao et al., 2012). Briefly, 100 μM of SP and OA were incubated for 30 min at 50 °C. Later, fatty acids were mixed with BSA in a culture medium (the fatty acid to BSA molar ratio was 4:1). To induce steatosis, HepG2 and Huh7 cells were exposed to SP and OA conjugated with fatty acid-free BSA. After incubation for 24 h, the cells were treated for 24 h with various concentrations of withaferin A (1, 2.5, and 5 μM). Cells used as controls were treated with fatty acid-free media containing ethanol as a vehicle.

### Oil Red O staining

Following treatment, the cells were fixed for 1 h in 10% formalin. The fixative solution was removed and rinsed with PBS once and then treated with 60% isopropanol for 15 s to facilitate the staining of neutral lipids. Cells were then incubated with a 6:4 diluted Oil Red O solution for 1 min and then washed with PBS to remove the excess stain before being counter-stained for 1 min with hematoxylin stain. Then, cells were washed with distilled water to remove excess stains. Pictures of the lipid droplets were taken using an inverted microscope.

### Oil Red O quantification

Oil Red O staining was measured semi-quantitatively after staining with Hematoxylin, washing with dH_2_O, and additionally washing with 60% isopropanol. The extracted Oil Red O stain was then treated with 100% isopropanol and gentle rocking. Then, the red color absorbance was measured at 492 nm.

### Molecular docking

The binding affinity of withaferin A with the LXR and FXR was assessed using AutoDock Vina (Eberhardt et al., 2021; Trott et al., 2010). The crystal structure of the LXR/FXR complexed with withaferin A was used as the target structure in the docking study. Water molecules were eliminated from the docking study and checked for prior attachment to the ligand (withaferin A) before being removed from the dimensional structure using version 2.4 of the PyMOL tool. Discovery Studio software, which offers near-able binding residues, was used to further visualize the favored possessions from the findings obtained from the docking technique. This verified the selected ligands’ ideal docking postures, and their binding affinities were recorded. Docking poses and score calculations were used to determine the binding affinity of withaferin A as a ligand with LXR and FXR.

### Statistical analysis

The one-way ANOVA (Bonferroni *post hoc* test) test was used for the data average value calculation of the results and for statistical analysis, where *p* < 0.05 was considered significant. The significance of different groups was expressed using mean ± SEM values. *p < 0.05, **p < 0.01, and ***p < 0.001

## Results

### Withaferin A inhibited diet-induced obesity

**FIGURE 1:**
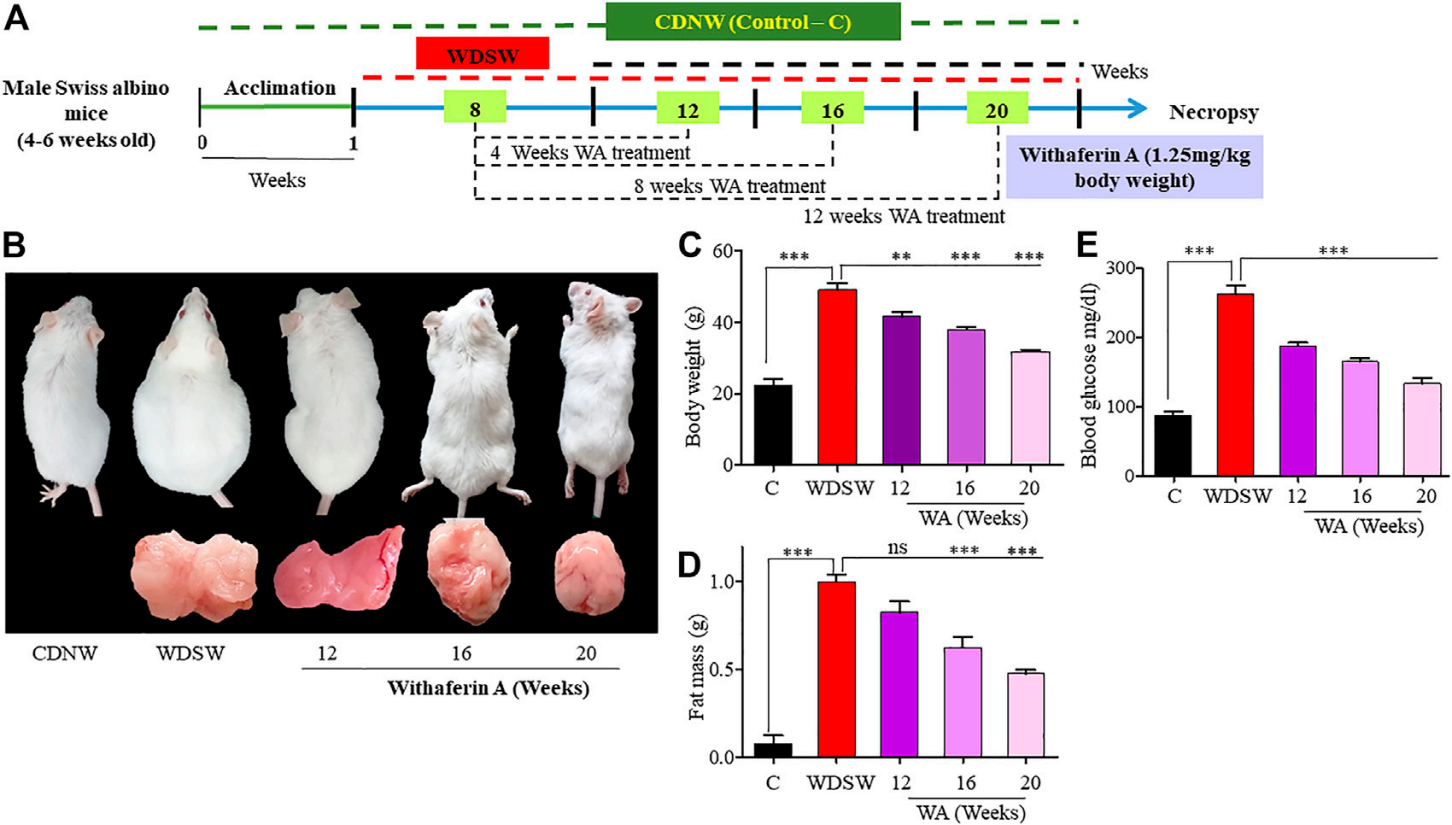
Withaferin A (WA) inhibited diet-induced obesity. **(A)** Experimental treatment pattern for testing the therapeutic effects of WA in the WDSW-induced NAFLD mouse model. **(B)** Images represent the reduction in body and adipose tissue weights in a withaferin A-treated WDSW-induced NAFLD mouse model. **(C,D)** Graphical representation depicting the decrease in body and adipose tissue weights in withaferin A-treated groups at different time intervals compared to the WDSW group. **(E)** Serum glucose levels. Data are expressed as mean ± SEM for six animals per group.

Swiss Albino mice nourished with WDSW showed advancement in weight and adipose tissue mass contrary to mice nourished with a chow diet and regular water (Figures 1A–D). In addition to progression in diet-induced obesity, nourishment with WDSW showed an elevation in blood glucose levels. Another group of mice fed with WDSW was treated with withaferin A for 12–16 weeks, which showed a considerable reduction in the total body weight (including fat mass) compared to WDSW-fed mice. Withaferin A also reduced blood glucose levels in mice with a high-fat diet and sugar water (Figure 1E). Our data are strongly supported by an elegant study from Harvard Medical School, which clearly shows the anti-obesity and anti-diabetic properties of withaferin A through its leptin sensitizing action, and it may also impact the appetite of the animal (Lee et al., 2016).

### Withaferin A decreased diet-induced liver injury and dyslipidemia

**FIGURE 2:**
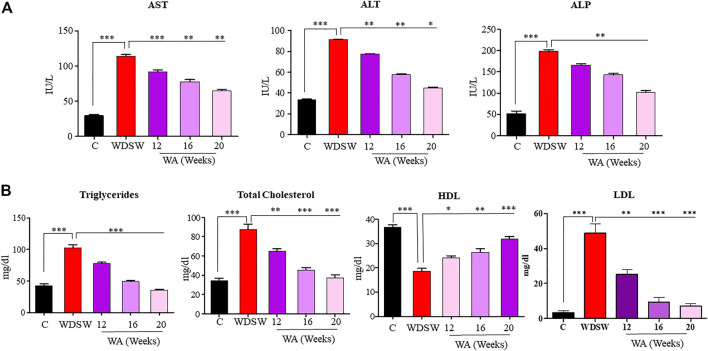
Withaferin A decreased diet-induced liver injury and dyslipidemia. Serum liver function tests: **(A)** aspartate aminotransferase (AST), alanine aminotransferase (ALT), and alkaline phosphatase (ALP). Serum lipid profile: **(B)** triglycerides (TG), total cholesterol (TC), high-density lipoproteins (HDL), and low-density lipoproteins (LDL).

Liver enzymes AST, ALT, and ALP increased in mice fed WDSW contrary to the control group (Figure 2A). The treatment of withaferin A reduced liver enzyme levels in different time courses. Withaferin A effectively lowered the lipid profile parameters in mice fed WDSW (Figure 2B). These data provide preliminary evidence showing the hepatoprotective effect of withaferin A.

### Withaferin A ameliorates steatosis and steatohepatitis both *in vivo* and *in vitro*

**FIGURE 3:**
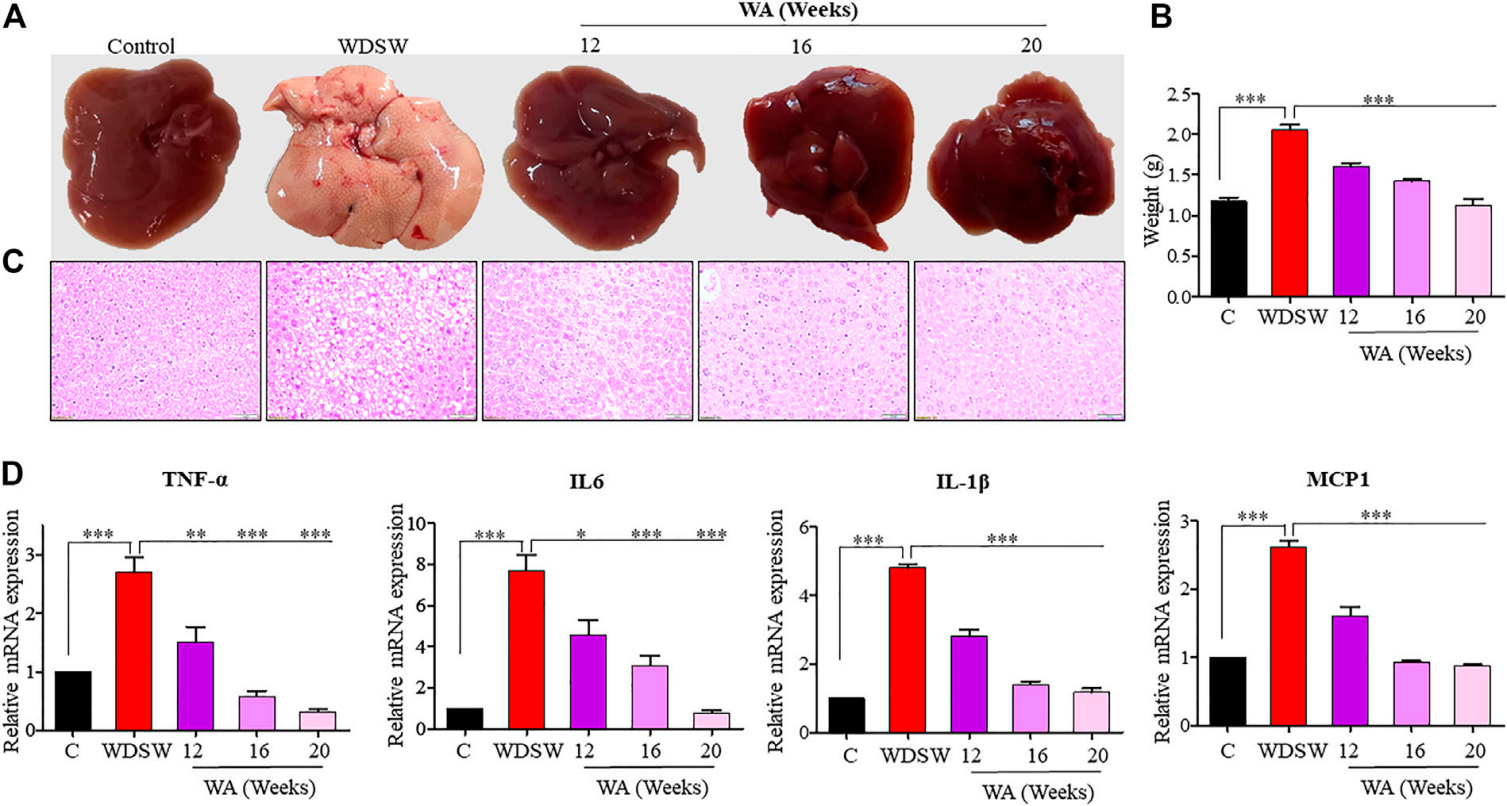
Withaferin A ameliorates steatosis and steatohepatitis both *in vivo*. **(A,B)** Representative images and graphs depicting a reduction in fat accumulation in the liver and liver weights in a withaferin A-treated WDSW-induced NAFLD mouse model. **(C)** Representative liver sections stained with hematoxylin-eosin (H&E) with a scale bar of 50 μM. **(D)** Relative mRNA expression levels of TNF-α, IL-6, IL-1β, and MCP1 were evaluated in the liver.

**FIGURE 4:**
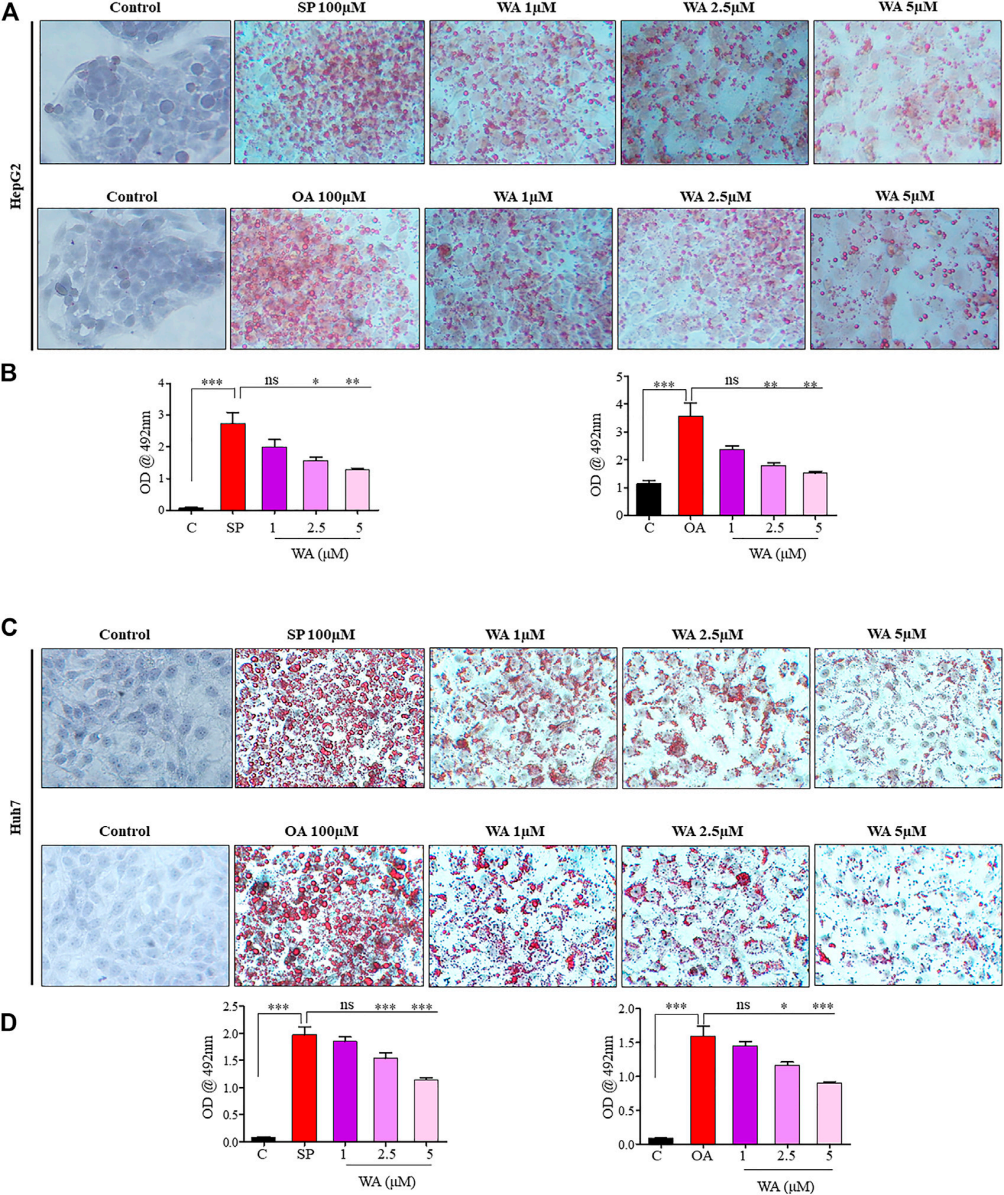
Withaferin A ameliorates steatosis and steatohepatitis *in vitro*. **(A,C)** Representative microphotographs of sodium palmitate and oleate-induced steatotic HepG2 and Huh7 cells treated with withaferin A in a dose-dependent manner. The cells were treated with the test materials for 24 h, and images were taken after ORO staining at 40X magnification. A semi-quantitative analysis of lipid accumulation in the cells **(B,D)**.

Mice nourished with a chow diet and regular water showed normal liver anatomy and weight (Figures 3A, B). In contrast, mice on WDSW developed major hallmarks of steatohepatitis (grade 3 macrovesicular steatosis, immune cell infiltration, and hepatocellular ballooning along with some microvesicular steatosis), as confirmed by H&E staining (Figure 3C). The withaferin A treatment also inhibited inflammatory markers, such as TNF-α, IL-6, IL-β, and MCP1 expression, in diet-induced obese mice (Figure 3D) compared to the high-fat diet-fed mice in our qPCR data. *In vitro* steatosis was confirmed by routine and common Oil Red O staining using HepG2 and Huh7 cells (Figures 4A, C). The withaferin A treatment inhibited sodium palmitate- and oleate-induced lipid droplet accumulation in human Huh7 and HepG2 cells, and the graphs depict a quantitative decrease of lipid accumulation in the cells **(** Figures 4B, D **)**. This experimental evidence strongly shows the anti-steatohepatitis effect of withaferin A.

### Withaferin A inhibited diet-induced fibrosis and fibrogenic signaling

**FIGURE 5:**
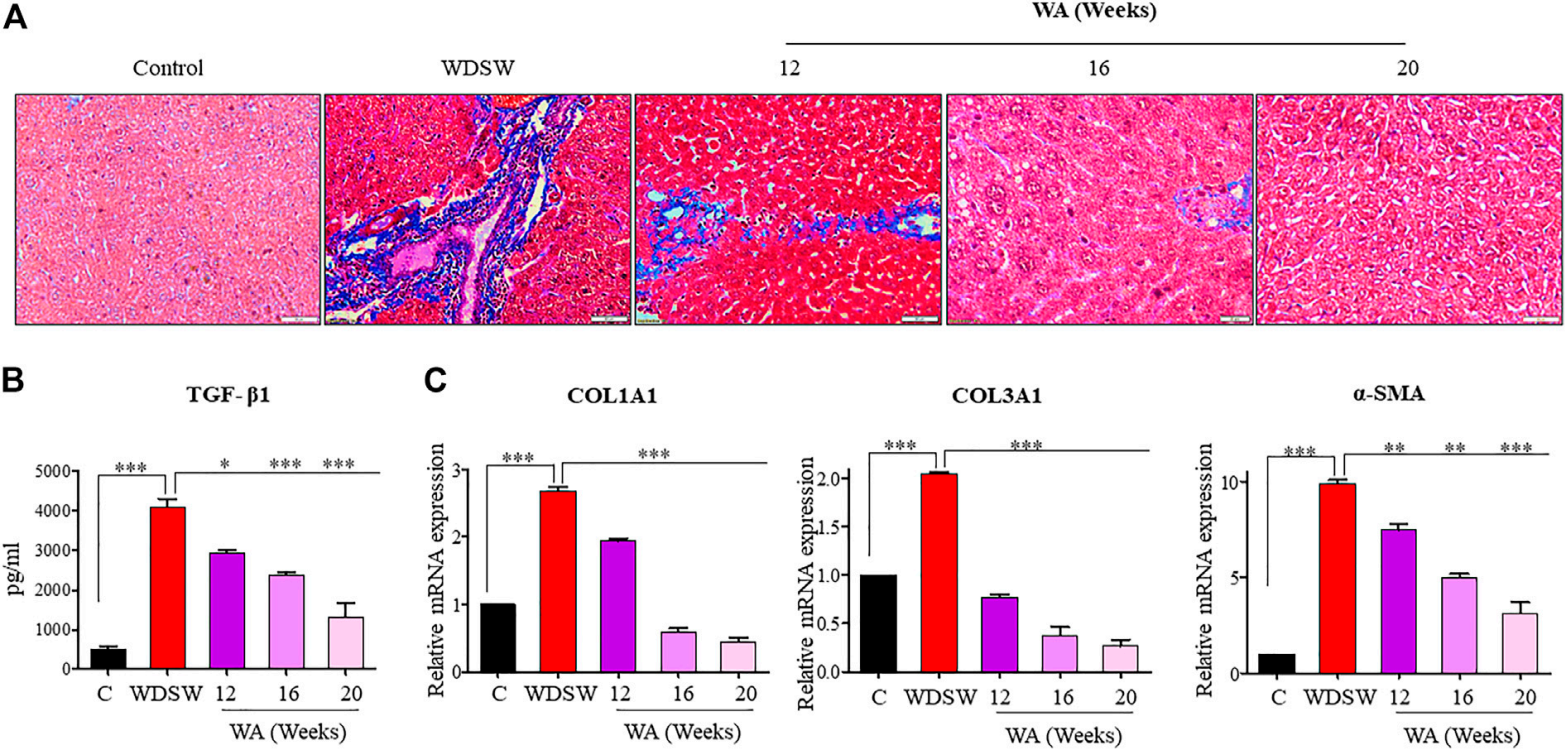
Withaferin A inhibits diet-induced fibrosis and fibrogenic signaling. **(A)** Representative liver sections stained with Masson’s trichome stain (MTS) with a scale bar of 50 μM. **(B)** Secretion of TGF-β1 by the liver in the withaferin A-treated WDSW-induced NAFLD mouse model measured by ELISA. **(C)** Relative mRNA expression of TGF-β1 target genes: *COL1A1*, *COL3A1*, and *α-SMA* were analyzed using qPCR.

Mice fed a regular low-calorie chow diet and normal water revealed normal liver architecture, whereas mice fed with high-calorie WDSW for 16–20 weeks revealed early fibrotic characteristics such as deposition of collagen and activation of TGF-β signaling. The withaferin A treatment inhibited diet-induced liver fibrosis in WDSW-fed mice (Figure 5A). The withaferin A treatment also inhibited TGF-β secretion and its target genes, Collagen 1 and Collagen 3, expression in WDSW-fed mice and was confirmed by ELISA and qPCR data (Figures 5B, C).

### Withaferin A revealed a dual LXR/FXR receptor-activating nature

**FIGURE 6:**
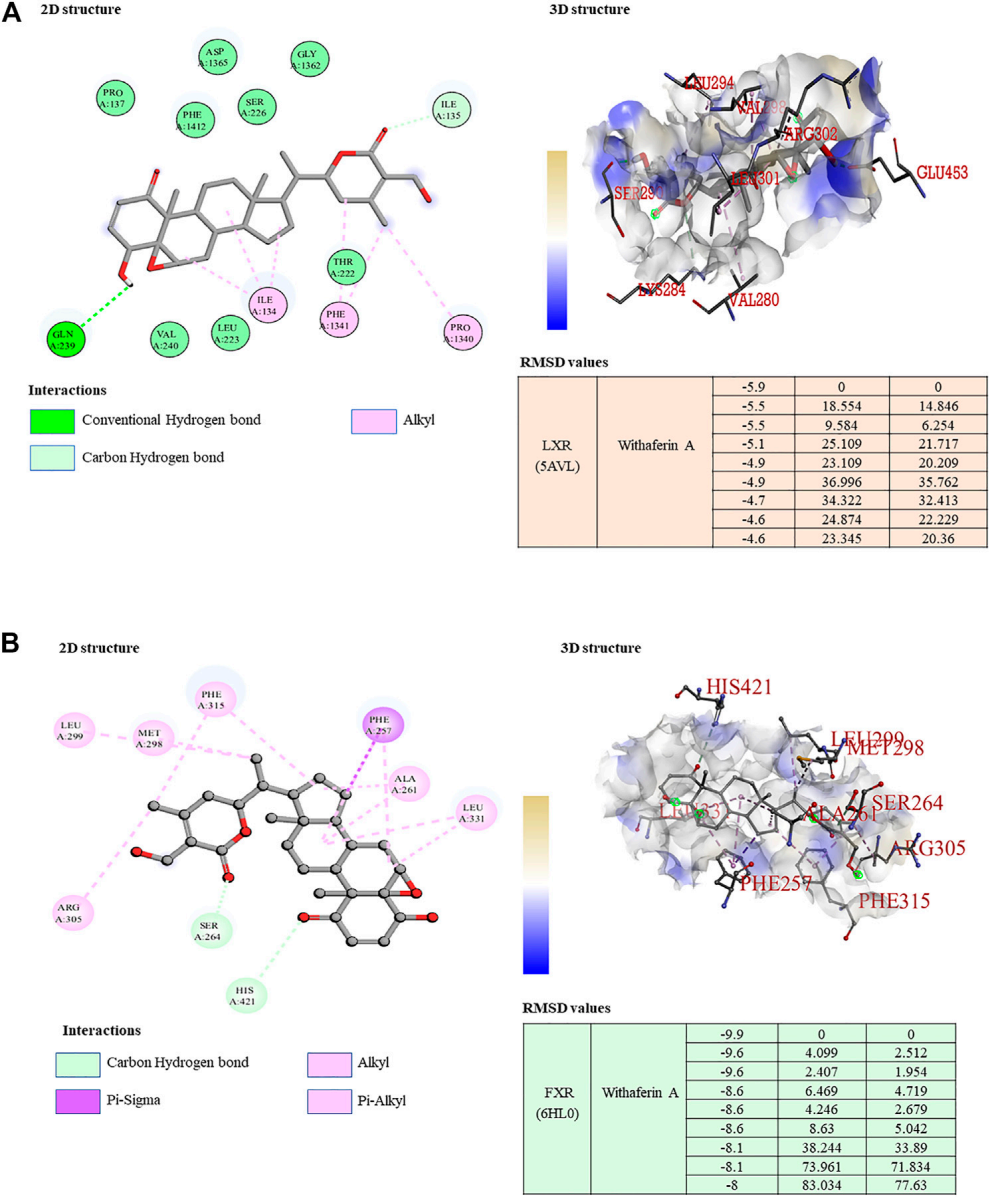
Withaferin A acts as a dual LXR/FXR receptor activator. **(A,B)** 2D and 3D visualization of the protein–ligand interaction of LXR **(A)** and FXR **(B)** with withaferin A; the table represents the binding affinity and RMSD values of the LXR/FXR receptor activator docked with withaferin A.

Our study revealed that withaferin A acts as a potent molecular ligand for LXR-α in HCC and inhibits NF-κB target genes (Shiragannavar et al., 2021; Shiragannavar et al., 2022). To determine the possible molecular mechanism of the hepatoprotective nature of withaferin A in the NAFLD model, we conducted a docking study and our results indicated a strong binding of withaferin A to both LXR-α and FXR, which is a known bile acid nuclear receptor (Figures 6A, B). Some of these common genes and signaling pathways are mutually regulated by ligand-dependent activation of both LXR-α and FXR (Ding et al., 2014), (Dong et al., 2019). Both inflammatory and fibrotic signaling is negatively regulated by LXR-α and FXR through their cognate ligands (Rudraiah et al., 2016; Wang et al., 2008). Based on our docking study results and the previously published evidence, we inferred that withaferin A acts as a dual LXR/FXR receptor activator and inhibits diet-induced steatosis, steatohepatitis, and fibrosis. To support our claim, we validated the expression of LXR-α and FXR and their target genes in both *in vitro* and *in vivo* NAFLD models treated with withaferin A. Along with withaferin A, we used the LXR-α specific agonist 25-hydroxycholesterol (25HC) and the FXR-specific agonist taurochenodeoxycholate (TCDC) and TGR5 (bile acid membrane receptor)-specific agonist deoxycholic acid (DCA). Our results showed that withaferin A activated both LXR-α and FXR and induced their canonical target genes (*ABCA1*, *ApoE*, *ABCB11*, and *ApoCII*) (Supplementary Figures S1–S3, S5, S7).

## Discussion

Obeticholic acid, which is a known agonist for FXR, has lately been under clinical trial for the management of NAFLD (Younossi et al., 2022). The molecular pathways for the therapeutic effects of obeticholic acid in FXR agonism are poorly understood and need to be elucidated. Another nuclear receptor, LXR-α, has a potential role in cholesterol homeostasis, and the ligand-dependent activation of LXR-α has an anti-inflammatory effect through repressing NF-ҡB-mediated signaling (Wu et al., 2009; Endo-Umeda and Makishima, 2019). Our study elucidated that withaferin A suppresses HCC proliferation, migration, and invasion *via* the activation of LXR-α and negatively regulates NF-ҡB target genes (Shiragannavar et al., 2021; Shiragannavar et al., 2022).

Although FXRs respond to bile acids and LXRs to oxysterol molecules inside the cellular nucleus, the ligand-specific coordinated actions of LXR and FXR activate transcription and modulate the expression profiles of several genes (Ding et al., 2014). In particular, genes are responsible for cholesterol, lipid, bile acid, and carbohydrate metabolism and control overall liver function (Dong et al., 2019). Along with their role in cellular metabolism, LXR and FXR activation also inhibits inflammation and fibrosis-associated gene expression through a transcription repression mechanism (Shiragannavar et al., 2021; Shiragannavar et al., 2022; Wang et al., 2008; An et al., 2020).

T0901317, a known LXRα [NR1H3] and LXRβ [NR1H2] agonist, also stimulates FXR more effectively than natural bile acid (Bonafide FXR ligand) and acts as a dual LXR/FXR agonist (Houck et al., 2004). However, T0901317 molecular action on pathophysiology remains elusive. There are reports that suggest T0901317 inhibits obesity and induces a fatty liver in mice (Gao and Liu, 2013a; Gao and Liu, 2013b). Also, numerous reports have shown the beneficial lipid-lowering effect of ligand-mediated FXR activation (Fang et al., 2015; Laffitte et al., 2003). Inflammation and fibrosis are major hallmarks of NAFLD-associated chronic conditions like HCC, where NF-κB acts as the master regulator of inflammation and inflammatory cytokine production (Elsharkawy and Mann, 2007; Santhekadur et al., 2012; Santhekadur et al., 2014). In addition, both LXR-α and FXR activation negatively regulates the activity of NF-κB (Wu et al., 2009; Shiragannavar et al., 2021; Wang et al., 2008).

Our docking studies clearly show that withaferin A acts as a bona fide ligand for both LXR-α and FXR and may activate both LXR-α and FXR and induce the expression of their target genes in NAFLD as a dual LXR/FXR receptor activator. In this study, we found the anti-obesity effect of withaferin A in diet-induced obesity mouse models and under *in vitro* steatotic conditions. FXR activation by its agonists promotes the browning of adipose tissue, induces thermogenesis, and reduces diet-induced obesity and insulin resistance (Fang et al., 2015). The activation of LXR improves glucose tolerance and plays an important role in regulating glucose metabolism in the liver and adipose tissue (Laffitte et al., 2003). This supports our data and shows the LXR/FXR dual receptor-activating nature of withaferin A and its therapeutic role in diet-induced obesity.

Overall, withaferin A treatment decreased ALP, AST, and ALT levels in diet-induced Swiss albino mouse serum in a time-dependent fashion in contrast to the WDSW-fed mice. Total cholesterol, non-HDL cholesterol, and circulating triglyceride levels were decreased in withaferin A-treated WDSW-fed mouse serum when compared with WDSW mice. Withaferin A also decreased hepatic triglyceride content in withaferin A-treated WDSW mice liver tissue when compared to WDSW mice (Supplementary Figure S8). This showed that withaferin A decreased diet-induced liver injury and dyslipidemia in Swiss albino mice. A few supporting studies have shown that FXR pharmacological activation prevents liver injury (Peng et al., 2021; Cui et al., 2009). Activation of LXRs also inhibits liver injury (Beyer et al., 2015). These studies strongly support our hypothesis that LXR/FXR dual activation prevents liver injury.

**FIGURE 7:**
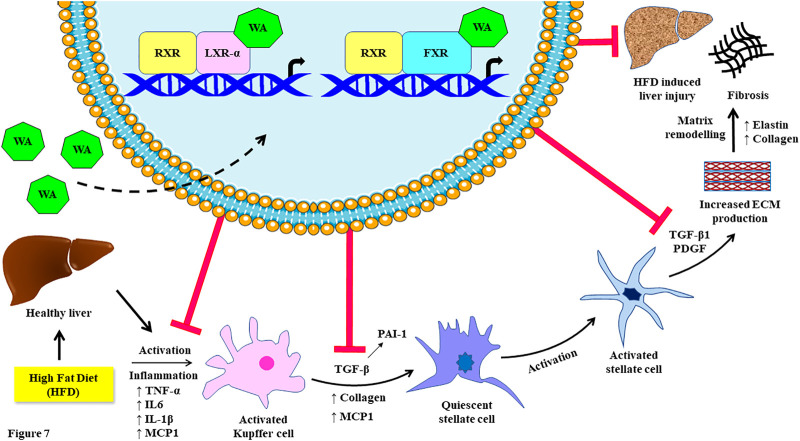
Schematic representation of the inhibiting effect of withaferin A on high-fat diet (Western diet sugar water)-induced fibrosis by acting as an LXR/FXR dual receptor activator. This figure was created in part using Servier Medical Art, which is licensed under a Creative Commons Attribution 3.0 Unported license.

We also found the anti-NASH and anti-fibrotic effects of withaferin A in our diet-induced NAFLD model (Supplementary Figure S4). It is already well-established and known that withaferin A has anti-inflammatory effects and inhibits NF-κB activation (Shiragannavar et al., 2021). The NF-κB activity is negatively regulated by LXR-α (Shiragannavar et al., 2022). Our immunohistochemistry data showed the anti-steatotic, anti-NASH, and anti-fibrotic effects in our diet-induced obese mice. Also, withaferin A inhibited IL-6, TNF-α, IL-1β, MCP1, COL1A1, COL3A1, and α-SMA expression in liver tissue. Additionally, past published studies have shown that LXR activation exerts a potent anti-inflammatory effect in immune cell types, particularly Kupffer cells/macrophages (Beyer et al., 2015). This may be due to the inhibition of MCP1 expression and suppression of Kupffer cell recruitment by withaferin A. Additionally, ligand-dependent LXR activation reduces acute hepatic inflammation, which is mostly mediated by macrophages that are unique to the liver (Kupffer cells) (Endo-Umeda and Makishima, 2019). LXR-deficient mice also revealed acute liver injury, steatohepatitis, and fibrosis due to excess hepatic cholesterol accumulation-mediated inflammation. Ligand-dependent LXR stimulation also suppressed primary stellate cell activation-mediated fibrosis. Additionally, Lxrαβ (−/−) stellate cells showed increased production and secretion of inflammatory mediators. The treatment of conditioned media from these Lxrαβ (−/−) cells to wild-type cells increased fibrogenic signaling and activated fibrosis (Beaven et al., 2011). A recent study demonstrated that the novel non-bile acid EDP-305 serves as a powerful and highly selective FXR agonist and potently inhibits the liver injury and fibrosis caused by a methionine/choline-deficient diet (An et al., 2020). It also inhibits NF-κB activity and suppresses the expression of TNF-α, IL-1β, COL1A2, COL3A1, α-SMA, and CCL2 (Chau et al., 2019). Another FXR agonist and known bile acid, obeticholic acid, protects against hepatic injury and fibrosis in a NASH mouse model (Goto et al., 2018). Withaferin A ameliorates bile duct ligation-induced liver injury and fibrosis by inhibiting NF-κB signaling (Sayed et al., 2019). Additionally, withaferin A therapeutically reduces fibrosis in HFD-treated leptin-deficient ob/ob mice (Sayed et al., 2019). In support of these classic studies, we found that withaferin A treatment inhibited fatty acid synthesis genes, such as sterol regulatory element binding protein 1c (SREBP1c) and fatty acid synthase (FASN), in both *in vitro* and *in vivo* NAFLD models (Supplementary Figure S3). It has been reported that the LXR-α agonist 25HC prevents NAFLD through the regulation of known cholesterol metabolism and inflammatory signaling (Wang et al., 2022). Increased levels of cholesterol 25-hydroxylase (Ch25 h) and its enzymatic by-product 25HC in the liver prevent high-fat diet-induced hepatic steatosis. This beneficial mechanism involves the regulation of enterohepatic circulation of bile acids by the induction of the *CYP7A1* gene *via* activation of LXR-α (Dong et al., 2022). Our data show that withaferin A also activates LXR-α and similarly induces its canonical target genes to that of 25HC and mimics and exerts similar effects on NAFLD. Previous studies show that hepatic overexpression of the canonical FXR target gene *ABCB11* reduces hepatosteatosis (Figge et al., 2004). Ligand-mediated FXR activation also ameliorates NAFLD mainly through a bile acid-dependent mechanism (Clifford et al., 2021). Interestingly, even in our study, the withaferin A treatment induced ABCB11 expression *via* FXR and mimicked the known FXR agonist taurochenodeoxycholate (TCDC) but not the TGR5-specific agonist deoxycholic acid (DCA) (Supplementary Figure S5). These experimental validations of withaferin A along with LXR-α- and FXR-specific agonists and previously published studies add strong support to our work. Based on our research, we propose a possible molecular mechanism involving the dual receptor-activating nature of withaferin A on LXR/FXR activation that ameliorates high-calorie diet-induced NAFLD (Figure 7).

In summary, we show that withaferin A not only activates LXR-α but also stimulates FXR, which is another similar nuclear receptor. Therefore, it exerts dual LXR and FXR ligand properties and confers protective effects against diet-induced animal models of obesity and NAFLD. These beneficial properties of withaferin A appear to be the consequences of decreased adipose tissue mass and glucose levels along with the hepatoprotective nature and anti-inflammatory and anti-fibrotic properties. Our data strongly suggest that the dual LXR/FXR ligand withaferin A might be used in the treatment of NAFLD and associated maladies.

## Data Availability

The original contributions presented in the study are included in the article/Supplementary Material, and further inquiries can be directed to the corresponding author.
