# Supplementary figures and images for: The ameliorating effect of withaferin A on high-fat diet-induced non-alcoholic fatty liver disease by acting as an LXR/FXR dual receptor activator
Source: Front Pharmacol. 2023 Feb 23;14:1135952. doi: 10.3389/fphar.2023.1135952 (PMC9995434; doi:10.3389/fphar.2023.1135952)

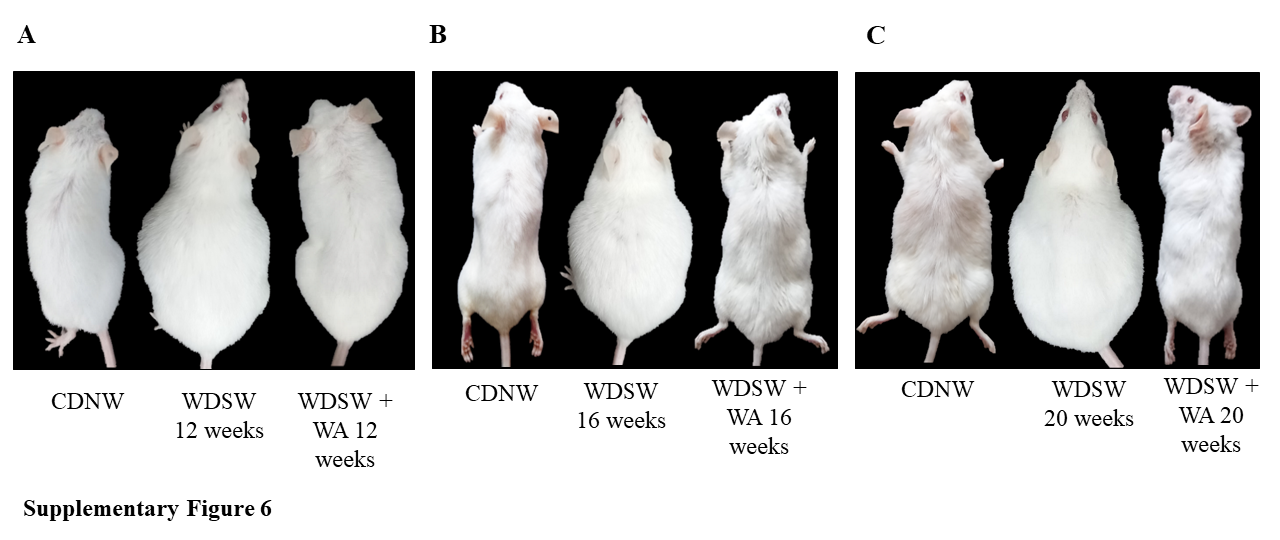

Supplement: Supplementary file 1 [file Image6.TIF]

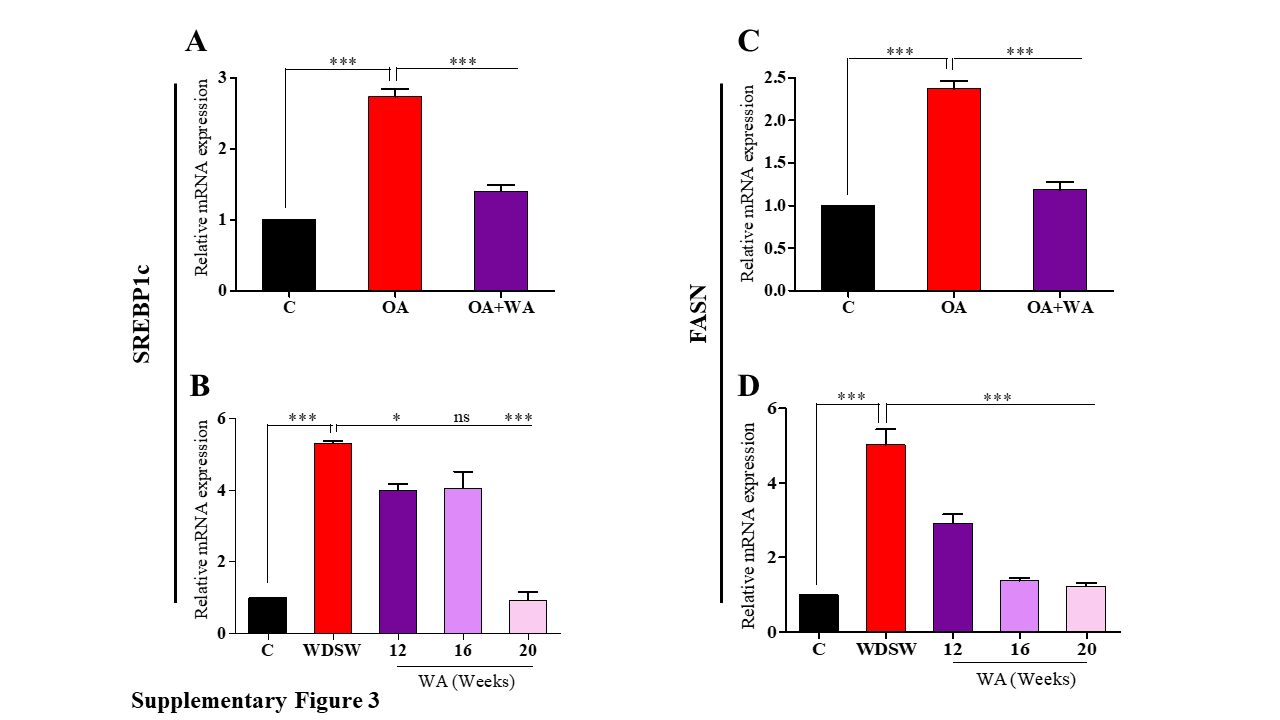

Supplement: Supplementary file 2 [file Image3.TIF]

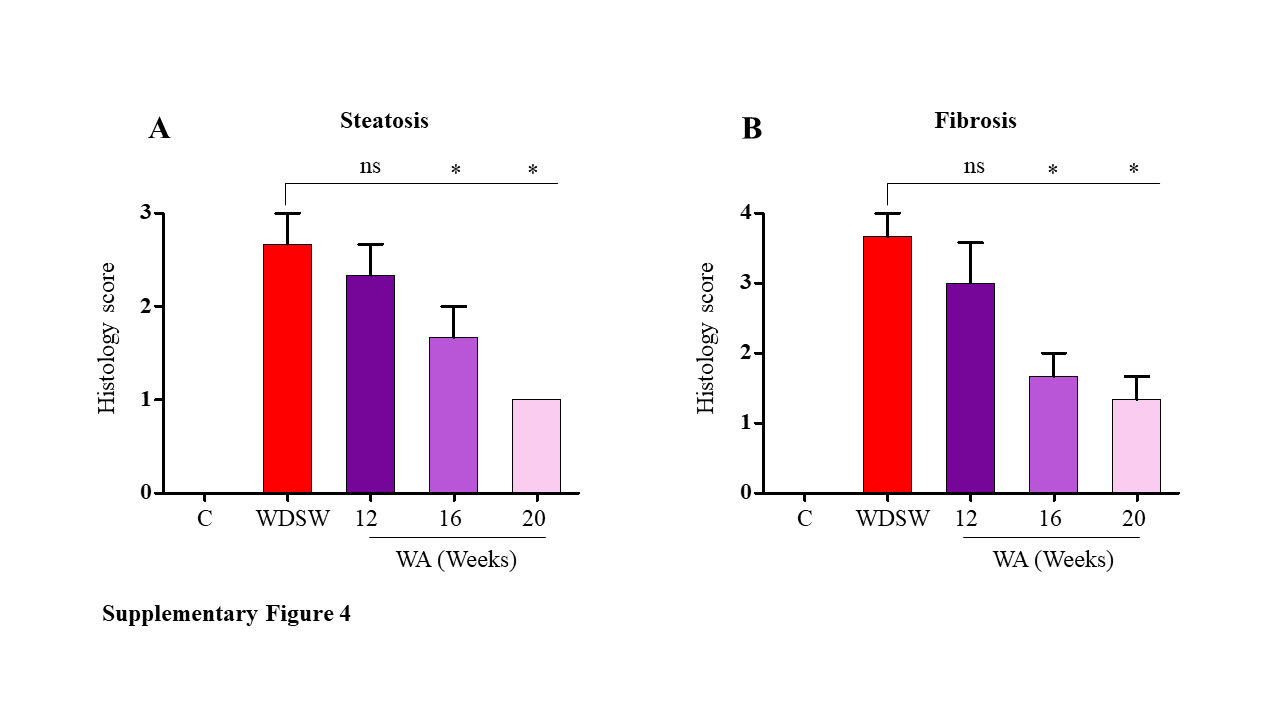

Supplement: Supplementary file 3 [file Image4.TIF]

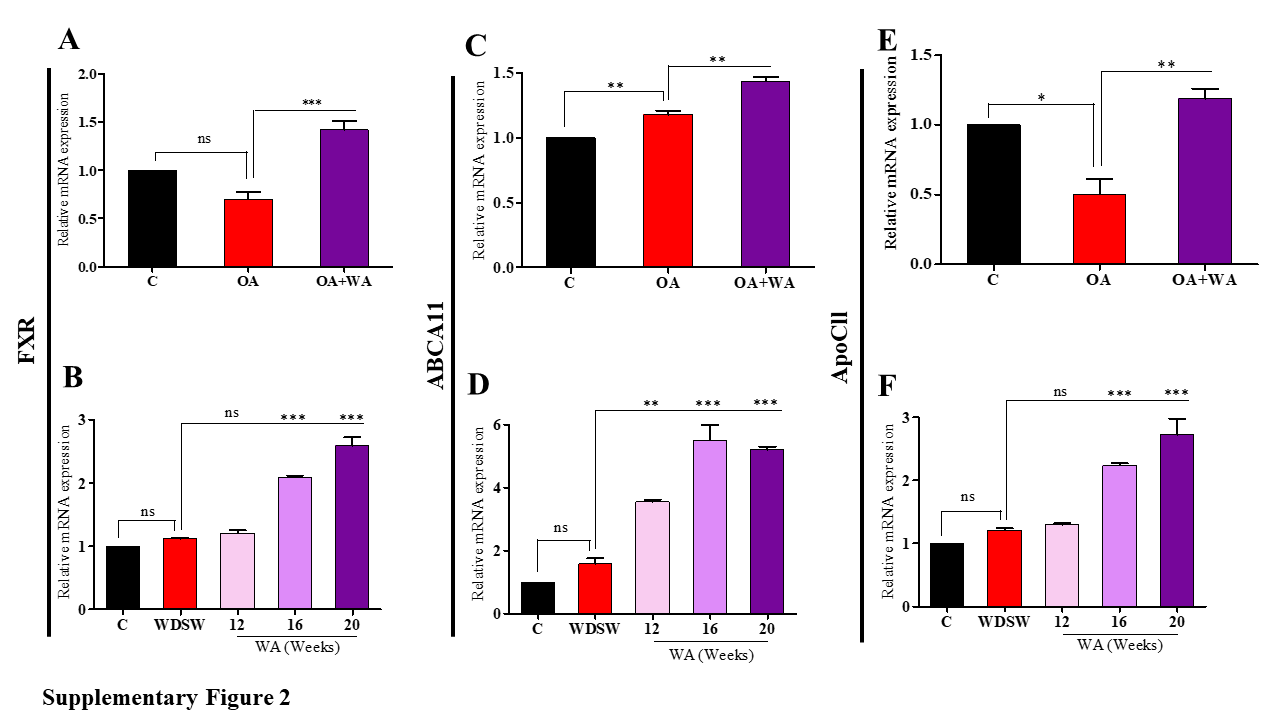

Supplement: Supplementary file 4 [file Image2.TIF]

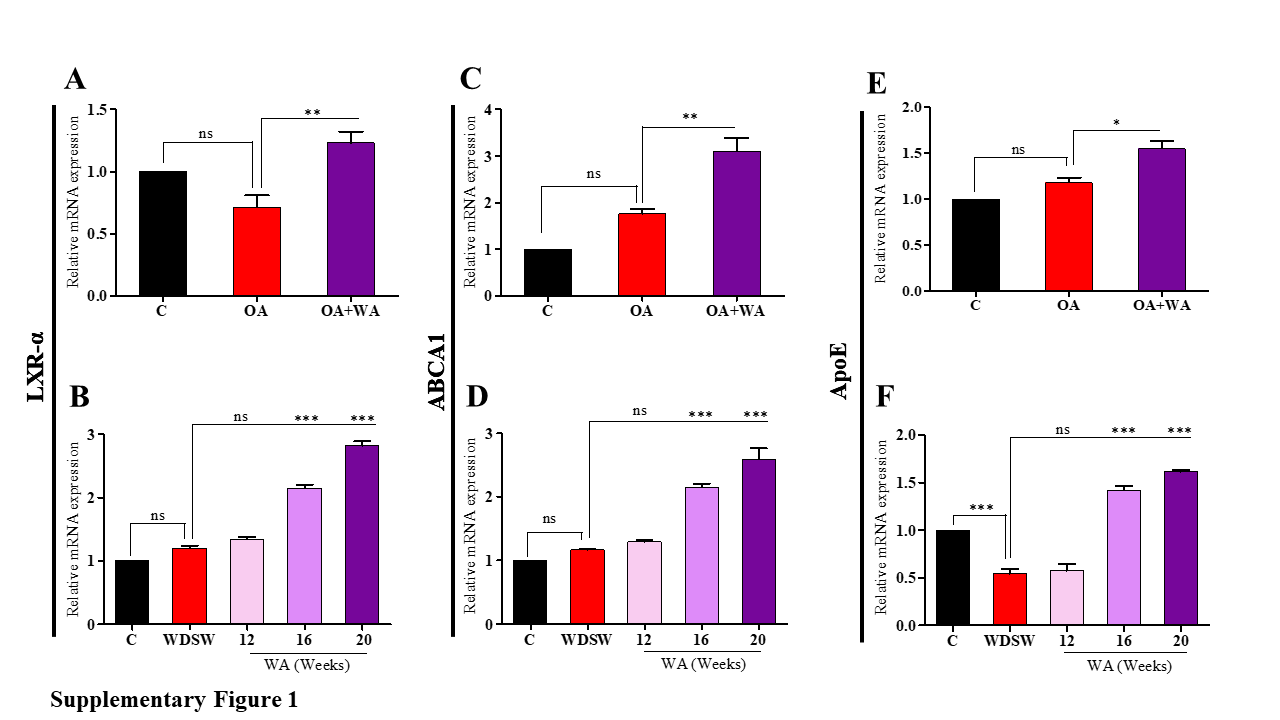

Supplement: Supplementary file 5 [file Image1.TIF]

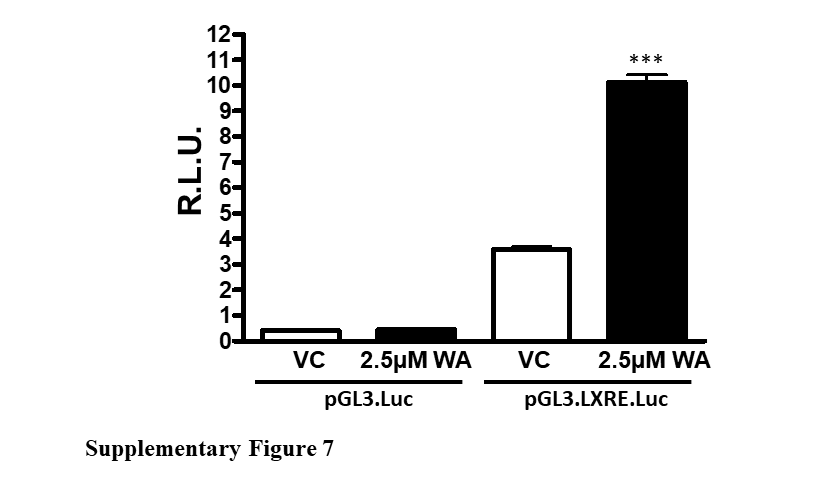

Supplement: Supplementary file 6 [file Image7.TIF]

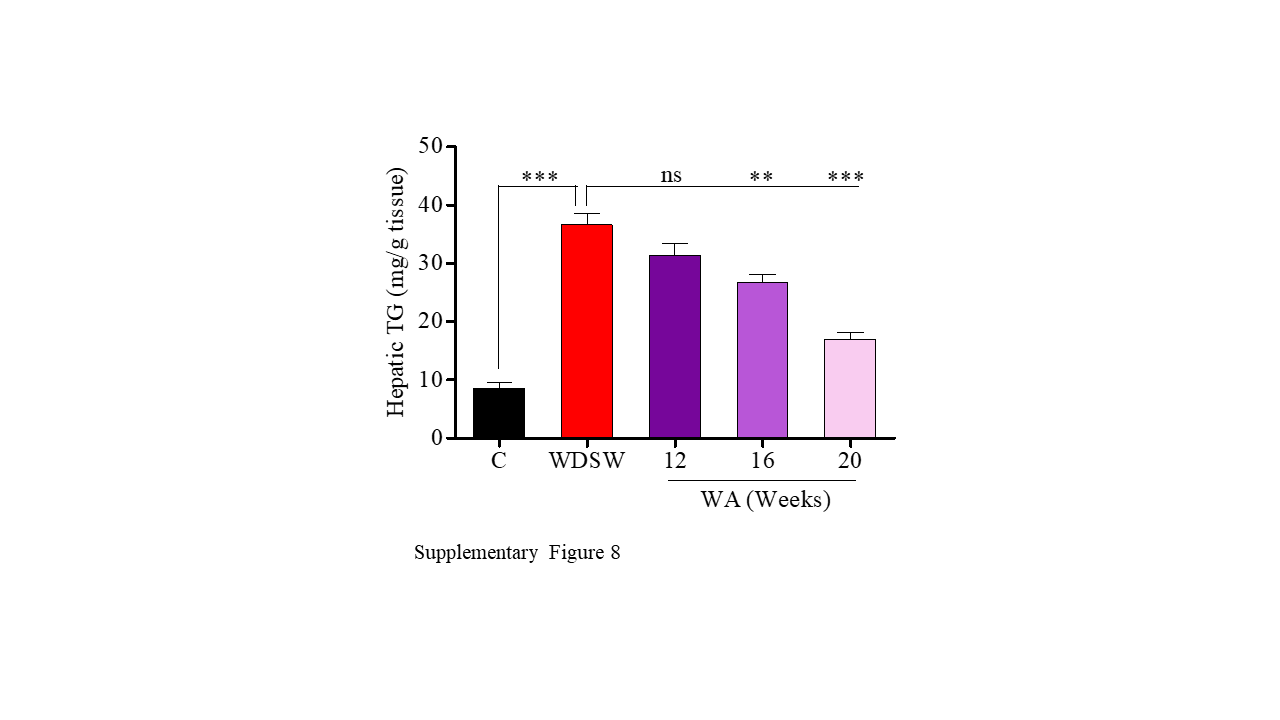

Supplement: Supplementary file 8 [file Image8.tif]

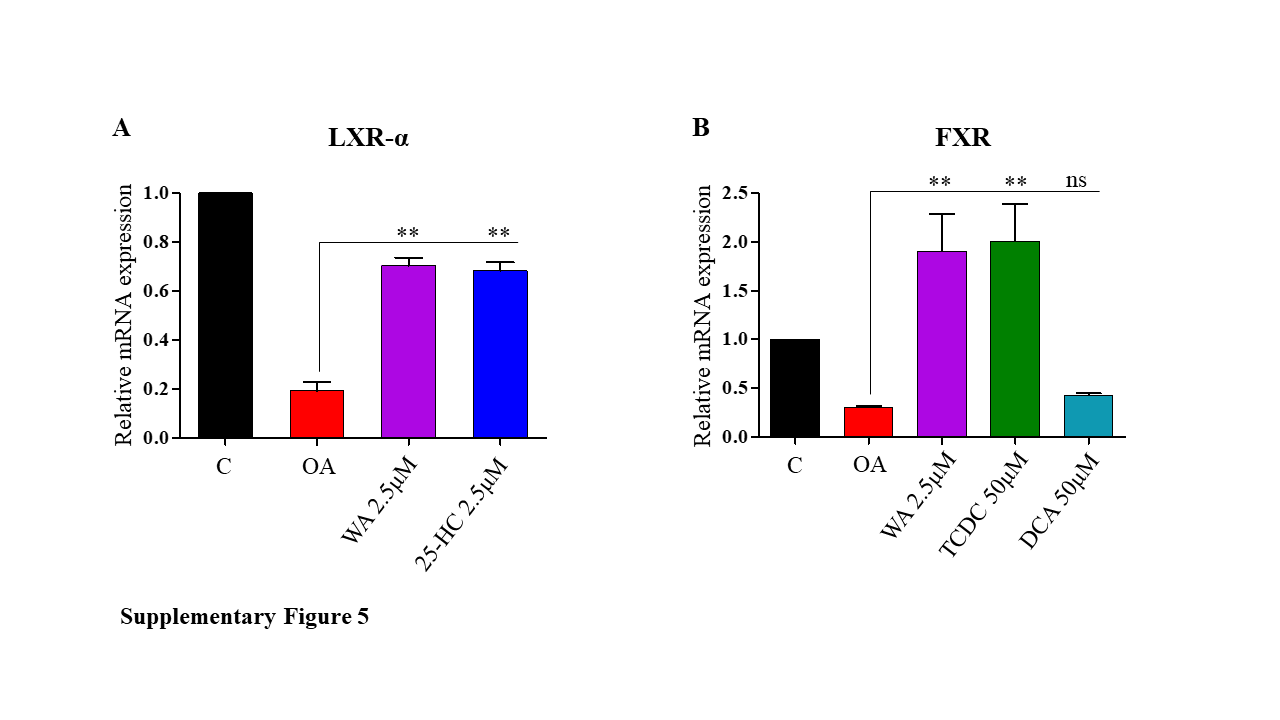

Supplement: Supplementary file 9 [file Image5.TIF]
