# Supplementary material for: The ameliorating effect of withaferin A on high-fat diet-induced non-alcoholic fatty liver disease by acting as an LXR/FXR dual receptor activator
Source: Front Pharmacol. 2023 Feb 23;14:1135952. doi: 10.3389/fphar.2023.1135952 (PMC9995434; doi:10.3389/fphar.2023.1135952)
Supplement: Supplementary file 7 [file DataSheet2.docx]

**Primer sequences**

(h - *Homo sapiens*, m – Mus musculus)

hLXR-α forward: 5’ – TGCCATCAGCATCTTCTCTG – 3’

hLXR-α reverse: 5’ – GGCTCACCAGCTTCATTAGC – 3’

hABCA1 forward: 5’ – TTCCCGCATTATCTGGAAAGC – 3’

hABCA1 reverse: 5’ –CAAGGTCCATTTCTTGGCTGT – 3’

hApoE forward: 5’ – GTTGCTGGTCACATTCCTGG – 3’

hApoE reverse: 5’ – GCAGGTAATCCCAAAAGCGAC – 3’

hFXR forward: 5’ – ACCAGCCTGAAAATCCTCAACAC – 3’

hFXR reverse: 5’ – CTCTCCATGACATCAGCATCTCAG – 3’

hABCB11 forward: 5’ – AGCCACACAGACCAGGATGTTG – 3’

hABCB11 reverse: 5’ – CAATGAACCGCCTCTCCTTTCC – 3’

hApoCll forward: 5’ – ACTGGGAGTCAGCAAAGACAGC – 3’

hApoCll reverse: 5’ – GCCTGTGTAAGTGCTCATGGCT – 3’

hSREBPc1 forward: 5’ – ACTTCTGGAGGCATCGCAAGCA – 3’

hSREBPc1 reverse: 5’ – AGGTTCCAGAGGAGGCTACAAG – 3’

hFASN forward: 5’ – ACATCATCGCTGGTGGTCTG – 3’

hFASN reverse: 5’ – GGAGCGAGAAGTCAACACCAA – 3’

mLXR-α forward: 5’ – GAGTGTCGATTCGCAAATGC – 3’

mLXR-α reverse: 5’ – CCTCTTCTTGCCGCTTCAGT – 3’

mABCA1 forward: 5’ – GGGCTCCTCCCTGTTTTTGA – 3’

mABCA1 reverse: 5’ – GTCAGCGTGTCACTTTCATGG – 3’

mApoE forward: 5’ – GAACCGCTTCTGGGATTACCTG – 3’

mApoE reverse: 5’ – GCCTTTACTTCCGTCATAGTGTC – 3’

mFXR forward: 5’ – GGGATGAGTGTGAAGCCAGCTA – 3’

mFXR reverse: 5’ – GTGGCTGAACTTGAGGAAACGG – 3’

mABCB11 forward: 5’ – CCTTGGTAGAGAAGAGGCGACA – 3’

mABCB11 reverse: 5’ – ATGGCTACCCTTTGCTTCTGCC – 3’

mApoCll forward: 5’ – GTTACTGGACCTCTGCCAAGGA – 3’

mApoCll reverse: 5’ – ATGCCTGCGTAAGTGCTCATGG – 3’

mSREBPc1 forward: 5’ – CAGACTCACTGCTGCTGACA – 3’

mSREBPc1 reverse: 5’ – GATGGTCCCTCCACTCACCA – 3’

mFASN forward: 5’ – CACAGTGCTCAAAGGACATGCC – 3’

mFASN reverse: 5’ – CACCAGGTGTAGTGCCTTCCTC – 3’
