# Supplementary material for: The ameliorating effect of withaferin A on high-fat diet-induced non-alcoholic fatty liver disease by acting as an LXR/FXR dual receptor activator
Source: Front Pharmacol. 2023 Feb 23;14:1135952. doi: 10.3389/fphar.2023.1135952 (PMC9995434; doi:10.3389/fphar.2023.1135952)
Supplement: Supplementary file 10 [file DataSheet1.docx]

**Supplementary figure legends:**

**Figure 1:** Withaferin A (2.5µM) upregulated LXR-α **(A)** and its target genes ABCA1 **(C)**, ApoE **(E)** in HepG2 cells. Similar pattern of gene expression was observed in Withaferin A treated mice liver samples for different time intervals **(B, D, F)**.

**Figure 2:** Withaferin A (2.5µM) upregulated FXR **(A)** and its target genes ABCA11 **(C)**, ApoCll **(E)**. Similar pattern of gene expression was observed in Withaferin A treated mice liver samples for different time intervals **(B, D, F)**.

**Figure 3:** Withaferin A (2.5µM) downregulated lipid metabolism associated genes SREBP1c **(A)** and FASN **(C)** in steatosis induced HepG2 cells. Withaferin A downregulated lipid metabolism associated genes SREBP1c **(B)** and FASN **(D)** in liver tissue at different time course.

**Figure 4:** Histopathology score analysis depicted augmentation in steatosis **(A)** and fibrosis **(B)** in H&E and TMS section respectively in Withaferin A treated groups over different time intervals.

**Figure 5: (A)** Withaferin A (2.5µM) upregulates LXR expression in a similar way to that of LXR agonist, 25-Hydroxycholesterol (25-HC: 2.5µM). (**B)** Withaferin A (2.5µM) upregulates FXR expression in a similar way to that of FXR agonist, Taurochenodeoxycholicacid (TCDC 50µM).

**Figure 6:** Representative images of CDNW, WDSW and Withaferin A treated mice groups for different time intervals (**6A** – 12 weeks, **6B** – 16 weeks, **6C** - 20 weeks).

**Figure 7:** Withaferin A transactivates LXR-responsive element (LXRE) via LXR-α in HepG2 cells

**Figure 8:** Graph representing hepatic triglyceride content depicting the lower levels of TG in liver when treated with Withaferin A for different time course.
